# BDNF and JNK Signaling Modulate Cortical Interneuron and Perineuronal Net Development: Implications for Schizophrenia-Linked 16p11.2 Duplication Syndrome

**DOI:** 10.1093/schbul/sbaa139

**Published:** 2020-10-17

**Authors:** Ashleigh Willis, Judith A Pratt, Brian J Morris

**Affiliations:** 1 Institute of Neuroscience and Psychology, University of Glasgow, Glasgow, Scotland, UK; 2 Strathclyde Institute of Pharmacy and Biomedical Sciences, University of Strathclyde, Glasgow, Scotland, UK

**Keywords:** 16p11.2 duplication, schizophrenia, CNV, critical period, CSPGs, parvalbumin

## Abstract

Schizophrenia (SZ) is a neurodevelopmental disorder caused by the interaction of genetic and environmental risk factors. One of the strongest genetic risk variants is duplication (DUP) of chr.16p11.2. SZ is characterized by cortical gamma-amino-butyric acid (GABA)ergic interneuron dysfunction and disruption to surrounding extracellular matrix structures, perineuronal nets (PNNs). Developmental maturation of GABAergic interneurons, and also the resulting closure of the critical period of cortical plasticity, is regulated by brain-derived neurotrophic factor (BDNF), although the mechanisms involved are unknown. Here, we show that BDNF promotes GABAergic interneuron and PNN maturation through JNK signaling. In mice reproducing the 16p11.2 DUP, where the JNK upstream activator *Taok2* is overexpressed, we find that JNK is overactive and there are developmental abnormalities in PNNs, which persist into adulthood. Prefrontal cortex parvalbumin (PVB) expression is reduced, while PNN intensity is increased. Additionally, we report a unique role for TAOK2 signaling in the regulation of PVB interneurons. Our work implicates TAOK2-JNK signaling in cortical interneuron and PNN development, and in the responses to BDNF. It also demonstrates that over-activation of this pathway in conditions associated with SZ risk causes long-lasting disruption in cortical interneurons.

## Introduction

Schizophrenia (SZ) is a devastating mental illness with positive (eg, delusions and hallucinations) and negative (eg, social withdrawal and anhedonia) symptoms combined with cognitive deficits.^1^ SZ is a neurodevelopmental disorder, with risk precipitated by genetic and environmental factors interacting to impact brain development.^2^Genetic risk involves multiple common variants of small effect. Rarely, single copy-number variants (CNVs) carry powerful genetic risk.^3,4^ The 16p11.2 duplication (DUP) is of special interest, as a high proportion of carriers develop SZ,^5,6^ and the effects are largely specific for SZ.^7^ 16p11.2 DUPs greatly increase the risk for SZ (OR = 10–30) while slightly increasing autism risk.^8–10^ The corresponding deletion clearly increases autism risk (OR = 5–20)^10^ and may slightly promote the risk of SZ-like symptoms.^4,11–13^

The 16p11.2 DUP region contains ~30 genes, including thousand and one kinase 2 (*TAOK2*) and mitogen-activated protein kinase 3 (*MAPK3*) (encoding the kinases TAOK2 and ERK1), both of which are implicated in neurodevelopmental disorders.^14–16^TAOK2 regulates mitogen-activated protein (MAP) kinase pathways, notably, the c-jun N-terminal kinase (JNK) pathway, where JNKs 1–3 are activated by upstream kinases mitogen-activated protein kinase kinase 4 (MKK4) or mitogen-activated protein kinase kinase 7 (MKK7).^17,18^ JNKs are strongly implicated in CNS development.^19–22^ Interest in JNK is strengthened by an association between variations in the *MAP2K7* gene (encoding MKK7) and prefrontal cortex (PFC) dysfunction in SZ.^23^

PFC gamma-amino-butyric acid (GABA)ergic parvalbumin (PVB) and somatostatin (SST) interneuron abnormalities represent a robust pathology in SZ.^24,25^ Associated with these interneurons are perineuronal nets (PNNs): complex mesh-like extracellular matrix structures enwrapping soma and proximal dendrites of, primarily, PVB-containing interneurons.^26^ These nets are dysregulated in SZ and in animal models.^27–29^ PNNs are involved in synaptic stability^30^ and plasticity,^31–33^ and their development is proposed to be synaptic activity dependent.^34^

Brain-derived neurotrophic factor (BDNF) promotes GABAergic maturation in the hippocampus and cortex.^35–37^ This is crucial for optimal development, as it leads to closure of the critical period of cortical plasticity,^38–40^ although the precise mechanisms are undefined. BDNF has become increasingly associated with SZ,^41^ with reduced levels of BDNF protein and messenger RNA (mRNA) in the patients’ blood.^42^ Considering the evidence for BDNF dysfunction in SZ, and its involvement in GABAergic interneuron development, we tested the hypothesis that JNK signaling might mediate these neurodevelopmental effects. Moreover, we investigated the potential disruption of cortical interneurons and PNN development in a 16p11.2 DUP mouse model and whether the same pathways might be involved.

## Methods

### 16p11.2 DUP Mice

The 16p11.2 DUP mice, which have been described previously,^43^ were obtained from the Jackson Laboratory and were maintained congenic on a C57Bl6/J background.

### Primary Cortical Neuronal Cultures

Primary cortical neurons were prepared using tissue from C57Bl6/J mouse embryos (E17), according to our standard procedures.^44^ For DUP cultures, females carrying the DUP were mated with wild-type (WT) males to produce WT and DUP embryos. Details of pharmacological treatments are in corresponding figure legends.

### Western Blot and Immunofluorescence

Protein was extracted from cultured neurons at 14 days in vitro (DIV) and processed for immunoblotting under a standard protocol.^45^ See supplementary table S1 for antibodies and dilutions in immunoblotting. Example images of full-length blots are available in supplementary figure S1.

For immunofluorescence, cells were fixed at 14 DIV with 4% paraformaldehyde for 30 minutes and incubated with anti-GAD65/67 and biotinylated lectin *Wisteria floribunda agglutinin* (WFA) (to detect PNNs) for 24 hours, with incubation in appropriate fluorescent secondaries for 1 hour; 8 DUP (4 female) and 8 C57BL/6 WT (4 female) littermates were transcardially perfused under terminal anesthesia with 4% paraformaldehyde, brains removed, and immersion fixed for a further 24 hours. After pairing by genotype and sex, 50 µm sections were incubated in 50% ethanol for 30 minutes, then with anti-PVB and biotinylated WFA for 48 hours, and then with secondary antibody for 24 hours. Full details of antibodies used can be found in supplementary table S1.

For cultured neurons, z stacks were obtained with a Biorad (MRC 1024) confocal microscope (scanning 8 µm planes, 0.5 µm steps), settings kept constant across related images. From 32 scans (GAD/WFA) from each treatment group (8 pairs/group), summed z stacks were analyzed. WFA staining intensity was measured using ImageJ. Optical densities were expressed as a percentage of vehicle control, unless otherwise stated. For tissue, 50 µm (0.5 µm steps) scans were obtained in pairs; 3 scans per region, and summed intensity z-stack projections quantified using ImageJ.

### Reverse Transcription-Quantitative PCR (RT-qPCR)

PFC tissue was obtained from paired adult WT and 16p11.2 DUP mice (10/genotype), and from postnatal day 14 (P14) WT and 16p11.2 DUP (8/genotype) mice. RNA was isolated using Qiagen RNeasy mini kits, and cDNA then synthesized (ThermoFisher), including noRT conditions. Primers (supplementary table S2) were validated for product size and specificity via gel electrophoresis. All amplifications were within 90%–110% efficiency. RT-qPCR reactions were performed using Fast SYBR Green (Applied Biosystems). Data were analyzed using ∆∆Ct method: *Gapdh* or *Tbp* was used as housekeeping genes, based on the best stability across all experimental conditions.

### Statistical Analysis

A complete table of statistics from all analyses can be found in supplementary table S3.

## Results

### BDNF Enhanced Cortical GABAergic Interneuron Maturation in a JNK-Dependent Manner

**Fig. 1.**
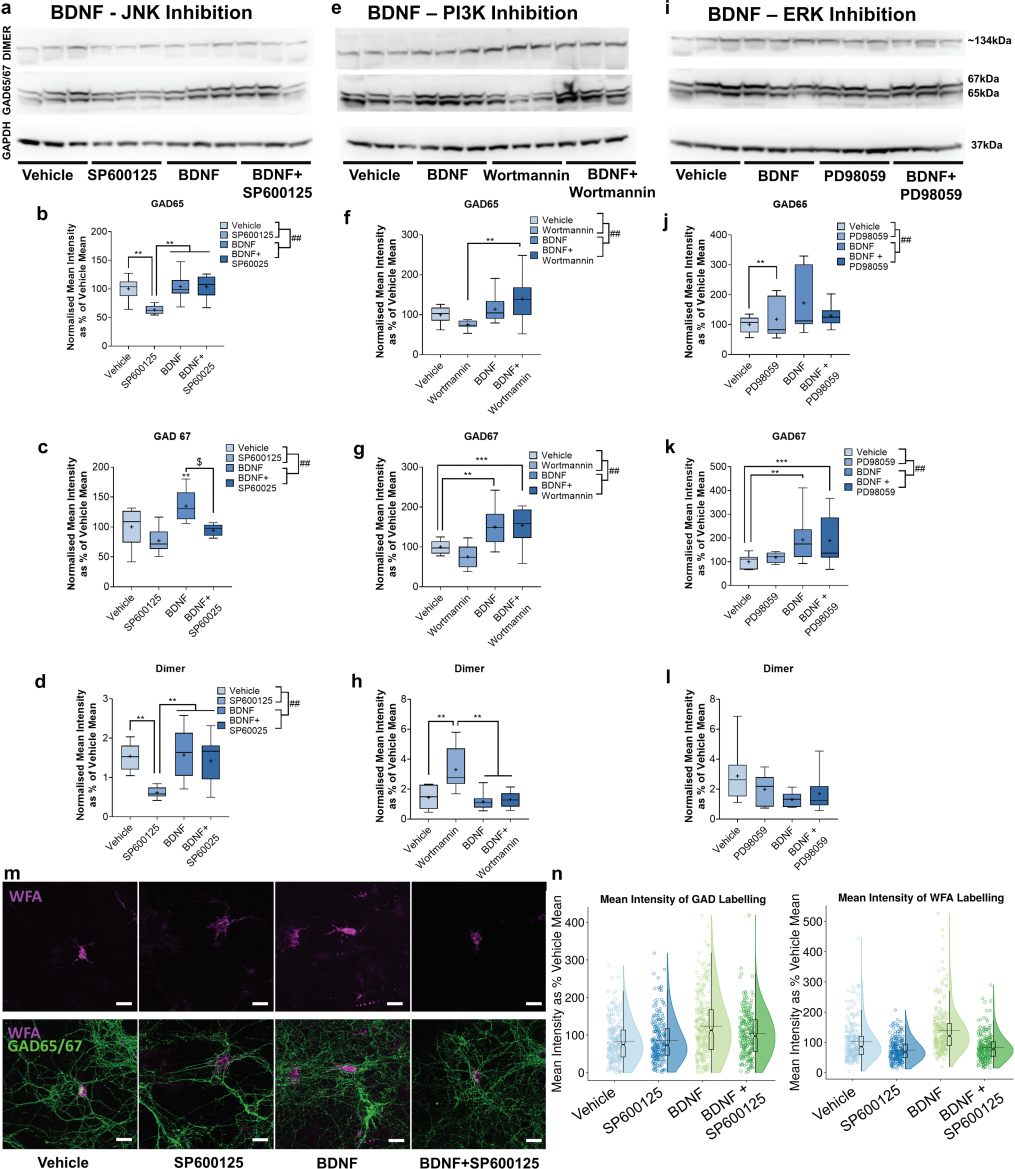
Brain-derived neurotrophic factor (BDNF) enhances GAD67 maturation in a JNK signaling-dependent manner. Western blot analysis of cortical neuronal cultures treated at 7 days in vitro (DIV) for 7 days with (a–d) vehicle, BDNF (50 ng/ml), JNK inhibitor (SP600125; 5 μM), or BDNF + SP600125. (e–h) vehicle, BDNF, PI3K inhibitor (wortmannin; 100 nM), or BDNF + wortmannin. (i–l) vehicle, BDNF, ERK inhibitor (PD98059; 40 µM) or BDNF + PD98059. *N* = 9 in all cases. BDNF increased GAD67 in all cases (c, g, k), but GAD65 and GAD dimer were generally unchanged (b, f, j, d, h, l). JNK inhibition negated the enhancing effect of BDNF on GAD67 levels (c). PI3K (g) and ERK (k) inhibition did not alter the response to BDNF. Data are presented as boxplots with medians, interquartile ranges, and “Tukey” whiskers; crosses indicate sample means. (m, n) immunocytochemical analysis of GAD65/67 and *Wisteria floribunda agglutinin* (WFA) labeling in primary cortical neuronal cultures treated with either vehicle, SP600125, BDNF, or BDNF + SP600125. *N* = 5 cultures (minimum 239 observations per condition). Representative images show WFA and merged stacked confocal imaged WFA + GAD65/67. Scale bars = 66 µm. WFA intensity was increased by BDNF treatment, but this effect was negated when JNK was inhibited. Raincloud plots show the probability density of immunocytochemical intensity data (ie, half violin) and notched boxplots, and horizontal lines plot the mean. Data were analyzed via 3-way ANOVA with factors BDNF, inhibitor, and culture. Factors BDNF and inhibitor were crossed to investigate any potential interaction. **P < .05, ***P < .01 Tukey post hoc significance vs indicated condition. $ indicates a significant interaction. The main effects are denoted by ## on graph legends. A full summary of *F* and *P*-values is contained in supplementary table S3 for all effects found in the analysis.

Primary mouse cortical cultures (14 DIV) were stimulated with BDNF to investigate their regulation of cortical GABAergic interneurons, along with inhibitors of the JNK signaling pathway (SP600125 [SP]), and 2 other well-characterized signaling pathways activated by BDNF-TrkB stimulation (ERK; PD98059 [PD], PI3K; wortmannin [WM]). We measured the levels of the GABA-synthesizing enzymes glutamic acid decarboxylase (GAD) GAD65/67 via Western blot and observed a clear enhancing effect of BDNF on GAD67 protein levels (figures 1a–d). This effect was replicated across all BDNF-TrkB inhibition experiments (figures 1e–l).

The accelerating effects on GAD67 maturation were negated when BDNF was administered in combination with the JNK inhibitor (SP). However, neither PI3K (WM) nor ERK (PD) inhibition attenuated BDNF-induced GAD67 increase. In fact, when BDNF was administered with PI3K (WM) or ERK (PD) inhibitors, GAD67 protein elevation remained or became further pronounced vs vehicle controls (figures 1g and 1k). JNK signaling is itself important to GAD protein expression; SP decreased the levels of GAD65 and GAD67 when administered to developing neuronal cultures.

The immunoblots also showed a higher weight (~134kDa) protein band that represents a GAD dimer^46^ (figures 1d, 1h, and 1l). SP reduced GAD dimer levels, while WM appeared to elicit an increase.

PNNs in vitro do not reach maturation until around 21 DIV,^47^ so are still developing at 14 DIV, but with net-like structures clearly present around the cell soma and proximal dendrites (figure 1m). BDNF increased the density of cell-body enwrapping PNNs (WFA staining intensity) (*P* < .001), an effect attenuated by JNK inhibition (figure 1n). Mirroring our above findings, SP alone reduced WFA intensity (*P* < .001).

**Fig. 2.**
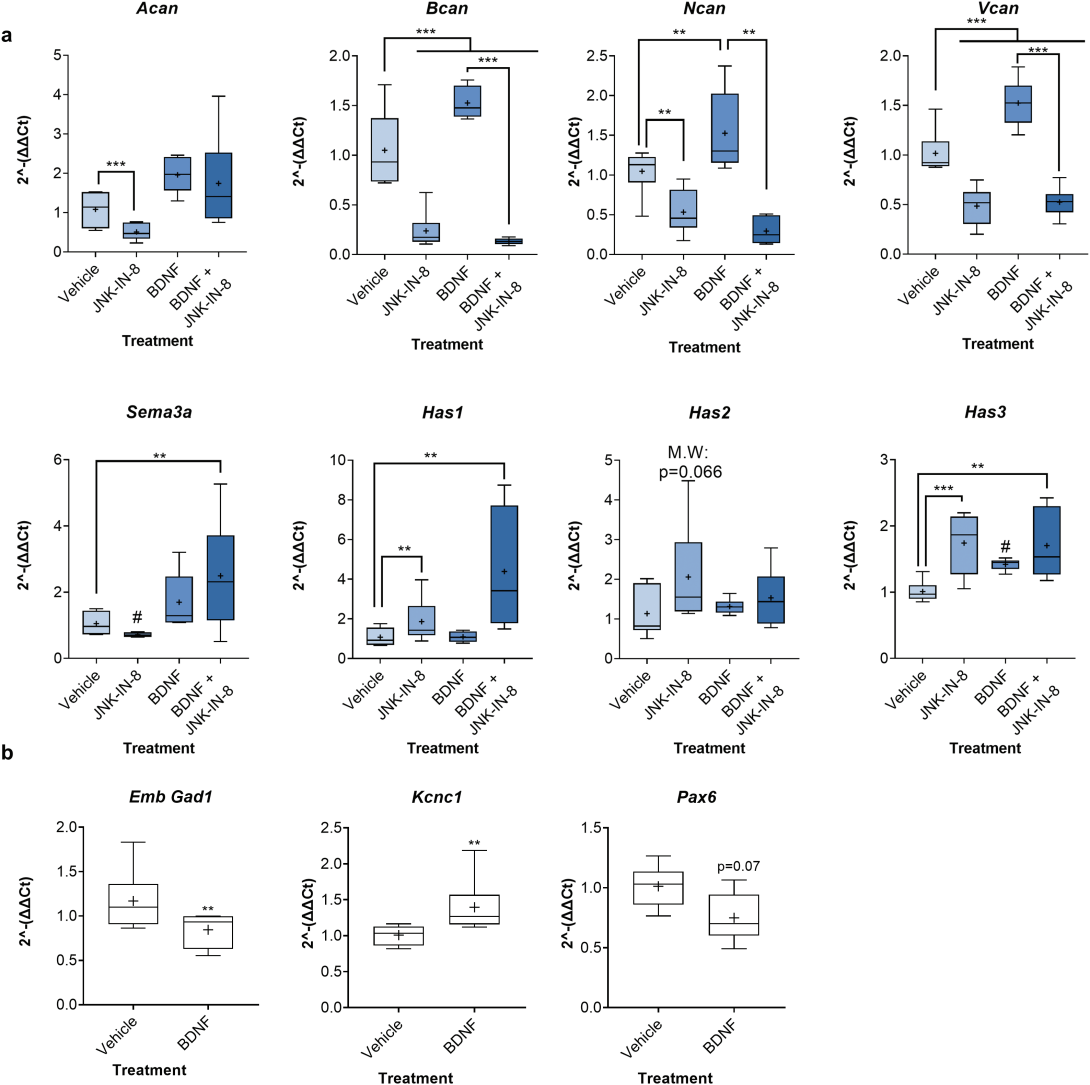
Brain-derived neurotrophic factor (BDNF) increases chondroitin sulfate proteoglycan (CSPG) (*Bcan, Ncan,* and *Vcan)* mRNA expression in a JNK-dependent manner and enhances the maturity of cortical neurons in culture. mRNA expression of key (a) perineuronal net (PNN) components (CSPGs, hyaluronan synthases [*Has1-3*], *Sema3a*) in cortical neuronal cultures treated with vehicle, JNK-IN-8 (1 μM), BDNF (50 ng/ml), or JNK-IN-8 + BDNF and (b) markers of cortical neuron maturity (embryonic *Gad1*, *Kcnc1,* and *Pax6*) were assessed in cortical neuronal cultures treated with vehicle or BDNF (50 ng/ml). All treatments were administered at 14 days in vitro (DIV) for 7 DIV (ie, RNA extracted at 21 DIV). The later time point chosen for inhibition (14–21 DIV) reflected the low levels or later developmental onset of some target genes and the subsequent requirement to have culture extracts containing high enough expression for qPCR detection. Relative expression was calculated using the ∆∆Ct method with *Tbp* housekeeping gene. Data are presented as boxplots with medians, interquartile ranges, and “Tukey” whiskers; crosses indicate sample means. Data (a) were analyzed via 2-way ANOVA (factors: BDNF, inhibitor) with Tukey post hoc multiple comparisons. Factors were crossed to investigate any significant BDNF-inhibitor interaction. **P < .05, ***P < .01 Tukey post hoc significance vs indicated condition. *Sema3a* and *Has2* vehicle vs JNK-IN-8 conditions were further analyzed via Mann-Whitney *U*-tests. # represents a significantly lower median expression of *Sema3a*, or a higher median expression of *Has3*, compared with vehicle. (b) Markers of cortical neuron maturity were analyzed via an independent sample *t*-test. ** represents significance vs vehicle (*P* < .05). *n* = 6 independent samples per condition.

While lectin labeling provides an excellent method of visualizing PNNs, they are composed of a complex combination of chondroitin sulfate proteoglycans (CSPG), link proteins, and other extracellular molecules.^48^ For a more detailed view of regulatory effects on PNN development, we treated cortical neurons with vehicle, JNK-Inhibitor-8 (JNK-IN-8), BDNF, or BDNF + JNK-IN-8^49^ and analyzed key PNN gene expression (figure 2a, supplementary figure S2).

Cultures treated with either vehicle or BDNF for 7 DIV at 14 DIV had higher expression of major PNN CSPGs, brevican (*Bcan)*, neurocan *(Ncan)*, and versican (*Vcan)*. Importantly, this BDNF-induced upregulation in CSPG expression was attenuated by JNK inhibition. Additionally, we found BDNF-induced upregulation of hyaluronan synthase 3 (*Has 3*), 1 of the 3 core enzymes responsible for the synthesis of the hyaluronan backbone of PNNs. Interestingly, this upregulation was not JNK dependent.

To further investigate whether BDNF accelerated cortical interneuron maturation, we measured the expression of a highly developmentally regulated *Gad1* transcript (Emb *Gad1*), which is expressed embryonically and declines throughout postnatal development to barely detectable expression in adulthood.^50–52^ We also measured *Kcnc1*, encoding Kv3.1b potassium channels that are enriched in fast-spiking PVB interneurons.^53,54^*Kcnc1* is also strongly developmentally regulated, with maximal expression observed in adulthood.^55^ Finally, we measured *Pax6* expression, a gene implicated in a range of cortical developmental processes.^56^*Pax6* expression is highest during embryonic and early postnatal development, after which it declines rapidly, becoming confined to remaining immature neurons in the dentate gyrus and olfactory bulb.^57^

BDNF treatment reduced the expression of Emb *Gad1* (with a trend toward *Pax6* reduction) while increasing *Kcnc1* expression (figure 2b). This pattern of gene expression, coupled with increased WFA labeling and CSPG expression, is indicative of a more mature GABAergic interneuron phenotype.

### Disruption to JNK Signaling Results in PNN Component Dysregulation

Expression of the 4 major CSPGs was substantially reduced with JNK signaling inhibition (figure 2a and supplementary figure S2). A similarly large reduction was found in *Sema3a* mRNA (figure 2a and supplementary figure S2); considering the binding between specific CSPGs and SEMA3A protein,^58^ it is intriguing that they change in synchrony. Conversely, the expression of *Has 1–3* showed substantial upregulation (figure 2a and supplementary figure S2).

### 16p11.2 DUP Neurons Develop Abnormally

The 16p11.2 DUP contains 2 critical MAPK pathway genes: *Taok2*, an upstream regulator of JNK, and *Mapk3* (ERK1). While abnormal dendritic phenotypes in cultured neurons carrying the 16p11.2 micro-DUP have implicated increased *Mapk3* expression as a major influence,^15^*Taok2* also functions in dendritic growth, spine/synapse development, and excitatory neurotransmission.^14^

#### MAPK Activity Is Elevated in 16p11.2 DUP Neurons.

**Fig. 3.**
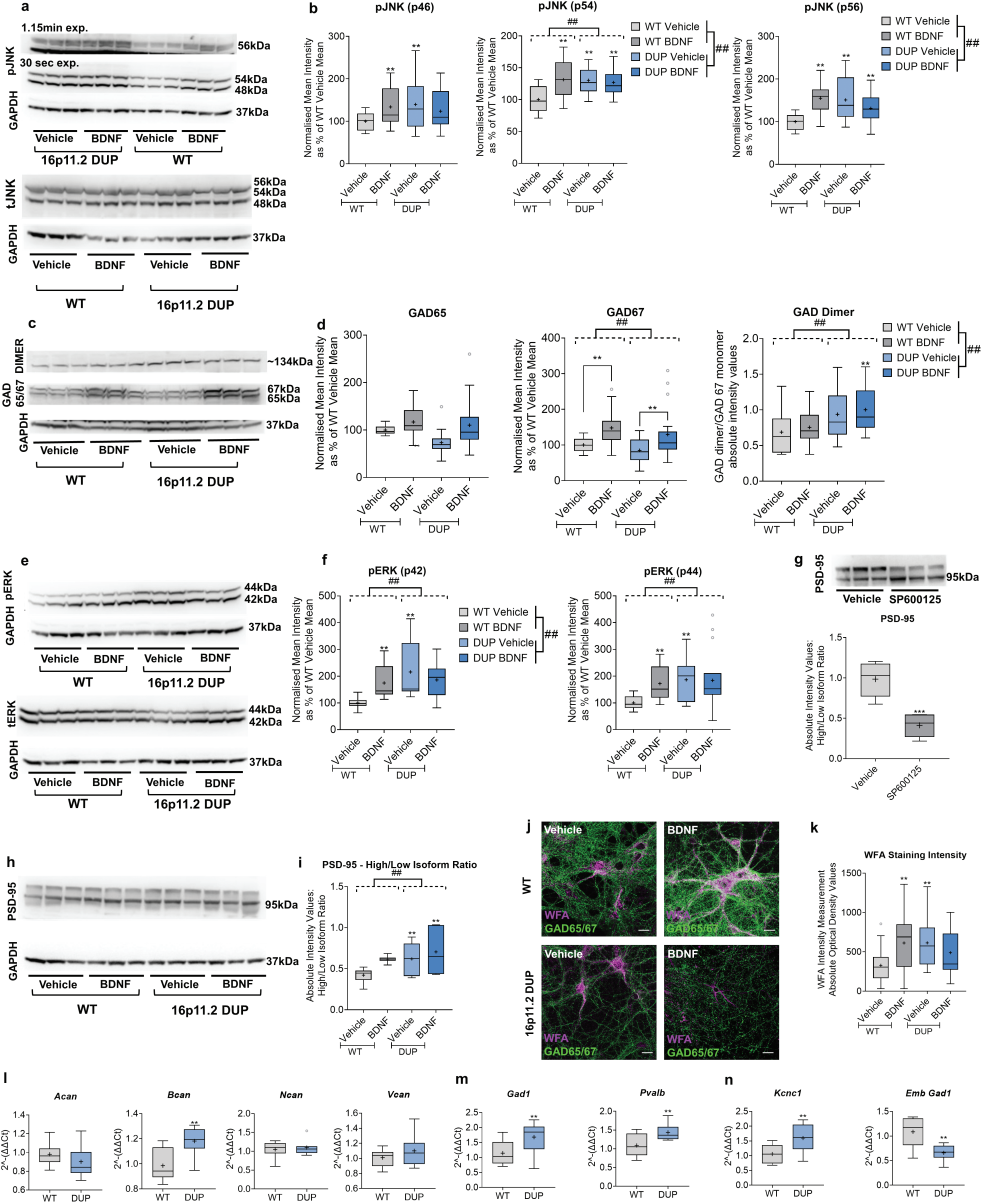
16p11.2 Duplication (DUP) neurons exhibit increased JNK activity, dysregulated GAD in vitro, and accelerated interneuron and perineuronal net (PNN) maturation in ex vivo postnatal prefrontal cortex (PFC). Primary neuronal cultures were obtained from 16p11.2 DUP and WT littermate embryos (E17) and were treated at 7 days in vitro (DIV) with either vehicle or BDNF (50 ng/ml) for 7 days. (a–g) Representative images and quantitative analysis of pJNK (a, b), GAD65/67 and GAD dimer (c, d), and pERK (e, f) in WT vs 16p11.2 DUP via Western blot. *N* = 18 (pJNK, tJNK, and GAD65/67), *N* = 9 (GAD dimer), and *N* = 15 (pERK and tERK) independent samples/condition, each of 3 independent cultures. (g) Representative images and Western analysis of specific JNK-phosphorylated form of PSD-95. *N* = 6 independent samples/condition, from each of 2 independent cultures. (h, i) This JNK-phosphorylated form of PSD-95 was found at increased basal levels in DUP cultures, compared with WTs via Western blot. *N* = 6 independent samples/condition, each of 3 independent cultures. (j, k) Immunofluorescent images and optical intensity analysis of cultured neurons from WT and DUP cultures treated with either vehicle or BDNF at 7 DIV for 7 days, labeled with WFA lectin and GAD65/67. DUP neurons had increased *Wisteria floribunda agglutinin* (WFA) staining intensity, which, unlike WT cultures, was unaffected by BDNF treatment. *N* = 18 independent samples/condition, each of 3 independent cultures. Minimum 8 confocal scans/independent sample, with at least 60 WFA measurements per condition. Scale bars = 54 µm. (l, m) mRNA expression of key CSPGs (l), *Gad1* and *Pvalb* (m), and markers of cortical neuron maturity, Kcnc1, and Emb Gad1 (n) were measured in samples of postnatal day 14 WT and 16p11.2 DUP PFC. *N* = 8 per genotype. For boxplots: Center lines show the medians, box limits indicate the 25th and 75th percentiles; whiskers extend 1.5 times the interquartile range from the 25th and 75th percentiles; crosses indicate sample means. O represents outliers. RT-qPCR data were analyzed via 1-way ANOVA. ** represents Tukey post hoc significance compared with WT vehicle (or as detailed via graph lines) (*P* < .05) and *** (*P* < .01). ## indicates the main effect of genotype (as indicated by dashed lines) or treatment (as indicated on graph legend).

We confirmed elevated JNK activity in DUP neurons, involving all 3 pJNK isoforms (figures 3a and 3b). In WT cultures, our data showed a clear elevation of pJNK 1–3 (figures 3a and 3b), and pERK (figures 3e and 3f), in response to BDNF. However, BDNF treatment of DUP culture did not elicit an increase in pJNK. This trend was the same in pERK levels, where DUP basal levels are already elevated vs WTs, and DUP pERK was unaffected by BDNF treatment.

#### GABAergic Markers and PNNs Are Dysregulated in 16p11.2 DUP Neurons.

DUP cultures exhibited dysregulated GAD, especially the 65-kDa isoform (figures 3c and 3d). Despite BDNF not clearly affecting the levels of the basally higher pJNK or pERK in DUP cultures, BDNF upregulated GAD67 levels comparably to WT cultures. This suggests that other downstream TrkB pathways are also involved in the effects of BDNF. Additionally, we found increased GAD dimerization in DUP cultures (figure 3d), the inverse of observations with JNK inhibition (figure 1d). These data support a role for JNK in functional GAD activity and suggest the dysregulation of GAD in 16p11.2 DUP neurons.

PSD-95 is an organizer of post-synaptic signaling complexes at glutamatergic synapses, with expression regulated according to the levels of synaptic activity.^59^ It is also regulated by phosphorylation via JNKs, which leads to an additional (more slowly migrating) immunoreactive band on Western blots.^60,61^ Therefore, we investigated the differences in PSD-95 phosphorylation/expression between WT and DUP cultures and observed 2 PSD-95 bands (figures 3g and 3h). To confirm the dependence of the more slowly migrating immunoreactive band on JNK activity, in WT cultures, as expected, SP produced a marked reduction in upper band intensity (figure 3g). BDNF enhanced the intensity of the higher weight, JNK-phosphorylated, form of PSD-95 protein in WTs (figures 3h and 3i). Basal levels of the upper isoform were markedly elevated in DUP cultures (figures 3h and 3i). Moreover, consistent with no increase in JNK activity in BDNF-treated DUP cultures, we found no further elevation of the high-weight PSD-95 isoform after BDNF treatment (figures 3h and 3i).

We observed an increase in DUP WFA staining intensity at 14 DIV (figures 3j and 3k). This may appear counterintuitive, considering the deficit in GABA synthesis. However, this finding aligns with the heightened JNK activity in these DUP cultures. Mirroring the trends in pJNK, BDNF did not increase WFA intensity in DUP cultures, but this effect was clear in WT cultures.

#### Postnatal 16p11.2 DUP Mice Exhibit an Interneuron Phenotype Indicative of Accelerated Development.

PNNs have a well-established role in development and synaptic plasticity, with denser or “tighter” nets being suggested as “brakes” on the developmentally timed restriction of cortical plasticity.^31,32,62–64^ Since DUP neurons exhibited denser PNNs in culture, we wished to explore whether this was indicative of an accelerated GABAergic interneuron developmental phenotype compared with WT mice.

We assessed the mRNA expression of key PNN CSPGs, mature *Gad1, Pvalb, Kcnc1*, and Emb *Gad1* in PFC samples from P14 WT and 16p11.2 DUP mice via RT-qPCR. We found a marked increase in *Bcan* expression in P14 DUP PFC compared with WT (figure 3l). Interestingly, *Bcan* expression changes have been linked to the closure of the critical period.^65^ Our analysis also revealed the higher expression of mature *Gad1, Pvalb*, and *Kcnc1* in DUP mice, while Emb *Gad1* was significantly reduced compared with WTs (figures 3m and 3n). These results indicate a clear acceleration in interneuron development and earlier PNN maturation in postnatal DUP mice.

### Adult 16p11.2 DUP Mice Have Sustained PNN Abnormalities and Interneuron Deficits in PFC and Thalamic Reticular Nucleus

**Fig. 4.**
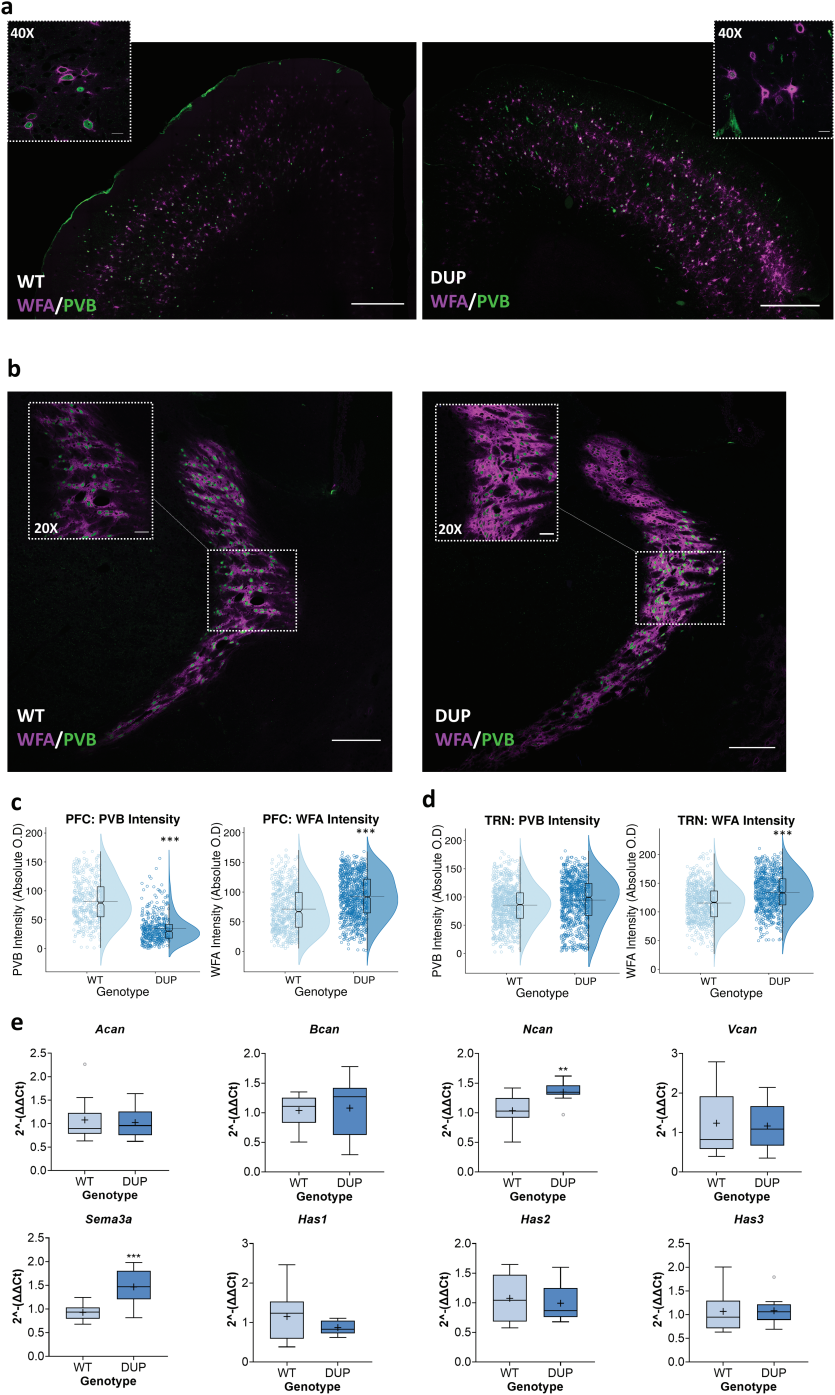
Perineuronal net (PNN) density is increased in adult 16p11.2 duplication (DUP) prefrontal cortex (PFC) and thalamic reticular nucleus (TRN), while PFC parvalbumin (PVB) levels are reduced. Labeling intensity of *Wisteria floribunda agglutinin* (WFA) lectin and PVB was measured in PFC and TRN of adult 16p11.2 DUPs and paired WTs. (a, b) provide representative *z*-stack images of PFC (a) and TRN (b). Raincloud plots of labeling intensity of PVB and WFA in the PFC (c) and TRN (d) indicate increased WFA intensity in DUP PFC and TRN vs WT. PVB was significantly lower in the PFC of DUP mice vs WTs, but unchanged in the TRN. Data were analyzed via 3-way ANOVA with factors genotype, sex, and pair, with factors genotype and sex crossed to investigate any interaction. *N* = 8 per genotype (4 male and 4 female). Minimum three 50-µm (20× obj. 0.5-µm steps) confocal scans analyzed per region, per mouse. Quantification was performed on summed intensity *z*-stack projections. Scale bars = 500 µm (PFC, tiled image), 20 µm (PFC, 40×), 200 µm (TRN, tiled image), and 50 µm (TRN, 20×). For raincloud plots: o is individual measurements, half violins represent probability density of data (ie, kernel density estimation), boxplots are notched on the median, and horizontal lines represent the mean. ***P < .01 represent Tukey post hoc significance vs WT. (e) Expression of key PNN components was measured in samples of adult WT and 16p11.2 DUP PFC. Relative expression was calculated using the ∆∆Ct method with *Gapdh* housekeeping gene. Data are presented as boxplots with medians, interquartile ranges, and “Tukey” whiskers; crosses indicate sample means. *N* = 10 per genotype (5 male and 5 female). RT-qPCR data were analyzed via 1-way ANOVA. **P < .05 and ***P < .01 represent Tukey post hoc significance vs WT.

We then investigated PNNs in adult mice carrying the DUP, focussing on 2 regions particularly pertinent to SZ, the PFC and the thalamic reticular nucleus (TRN), which are anatomically and functionally closely connected with the PFC, and where dysfunction is linked to SZ etiology.^66,67^ Additionally, given the link between PVB deficits and SZ, we monitored PVB levels via immunofluorescence intensity. WFA intensity was significantly increased in DUP compared with WT mice, indicating denser PNNs in these regions (figures 4a–d). Data for PVB are more nuanced. PVB intensity levels in TRN tended toward increased levels in DUPs. However, in PFC, we observed a clear reduction in PVB levels vs WTs.

Conducting a finer grain analysis of PNN components via RT-qPCR (figure 4e), the 2 major PNN components with increased expression in DUP vs WT PFC were *Ncan* and *Sema3a* (figure 4e). Considering the importance of PNNs in plasticity, we also investigated whether these processes may be perturbed in DUP mice, using the immediate early gene markers (IEGs) *cFos* and *Arc*. Expression of *cFos* was elevated in DUPs, with evidence of a genotype-sex interaction (supplementary figure S3).

### TAOK and ERK Inhibition Disrupt PNN Regulation and Interneuron Maturation in Developing Cortical Neurons In Vitro

**Fig. 5.**
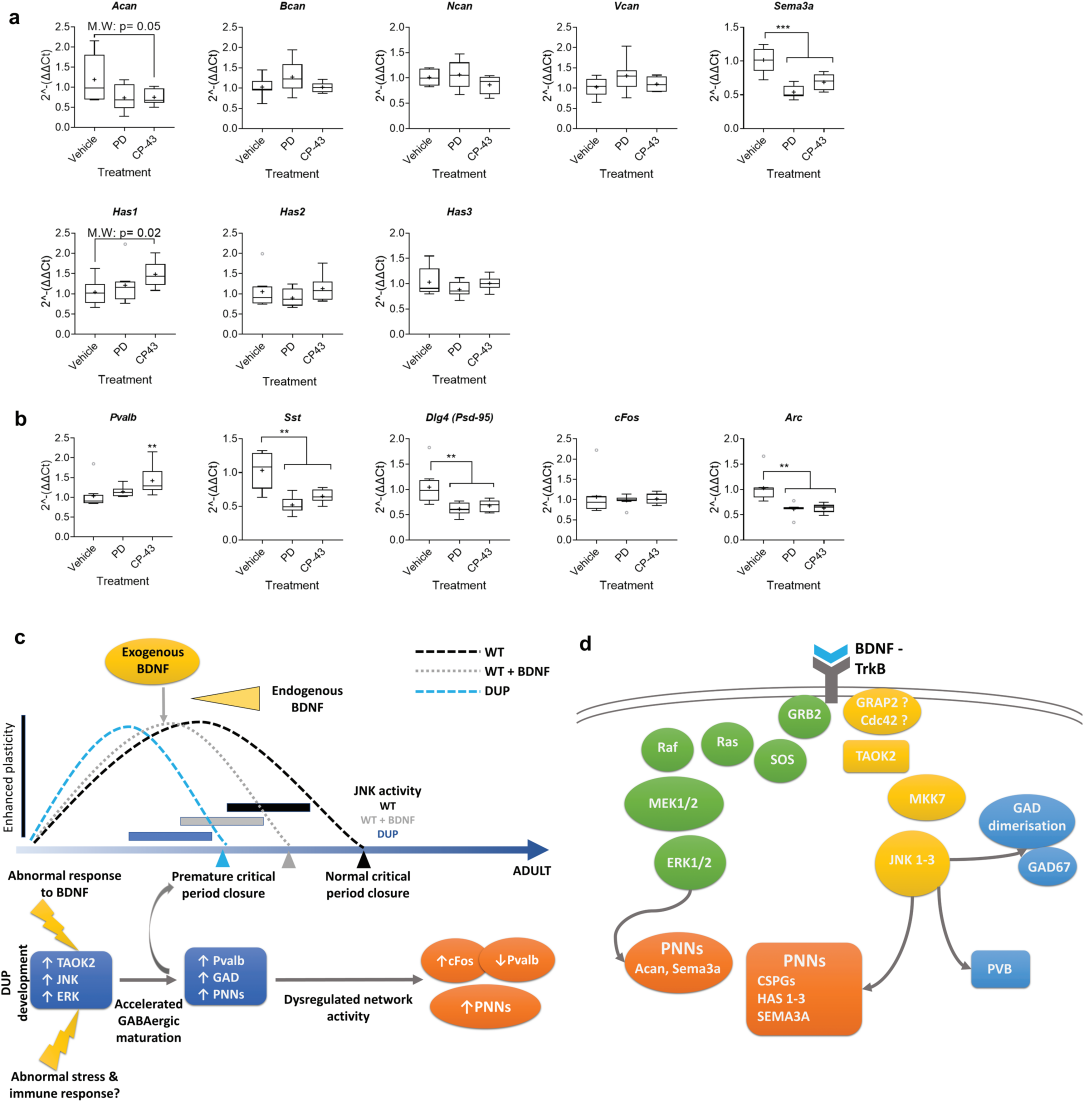
Pharmacological inhibition of TAOK and ERK disrupts the expression of key perineuronal net (PNN) and GABAergic genes and model of abnormal development in 16p11.2 duplication (DUP) neurons. Expression of key (a) PNN genes, (b) *Pvalb*, *Sst*, and *Dlg4* (PSD-95), and immediate early genes (IEGs: *cFos* and *Arc*) in WT cultures treated at 16 days in vitro (DIV) for 5 days with either vehicle, CP-43 (TAOK inhibitor; 30 μM), or PD (ERK inhibitor; 40 μM) was measured via RT-qPCR. Relative expression was calculated using the ∆∆Ct method with *Tbp* housekeeping gene. Data are presented as boxplots with medians, interquartile ranges, and “Tukey” whiskers; crosses indicate sample means. *N* = 8 independent samples per condition. Data were analyzed via 1-way ANOVA. Additionally, a Mann-Whitney *U*-test was performed for vehicle vs CP-43-treated samples on *Acan* and *Has1* data, where we found the median expression of *Acan* and *Has1* to be significantly lower or higher, respectively, in CP-43 samples (*P* = .05 and *P* = .02, respectively). **P < .05; ***P < .01 (Tukey post hoc significance). (c) 16p11.2 DUP neurons exhibit an abnormal developmental phenotype that may involve a premature closure of the critical period, as supported by elevated *Wisteria floribunda agglutinin* (WFA), *Ncan*, *Kcnc1*, mature *Gad1*, and *Pvalb* expression, and downregulated embryonic *Gad1*. Considering the data presented here, this is likely to be driven by *Taok2* over-expression and subsequent over-activity of JNK. Given the importance of parvalbumin (PVB)+ interneurons in the regulation of excitatory/inhibitory balance within cortical regions, accelerated GABAergic development may lead to dysregulated network activity and synaptic connectivity, potentially evidenced by an elevation in *cFos* in adult DUP prefrontal cortex (PFC). *Pvalb* expression is reduced in adult DUP PFC, which may indicate a compensatory mechanism to achieve network balance. Heightened PNN density is sustained into adulthood in DUP mice. Elevated JNK activity in developing DUP neurons may underlie sustained increased PNN density. Notably, we found that DUP and WT neurons respond differently to exogenous BDNF application in culture; JNK and ERK activity are not upregulated and there is no apparent BDNF-induced acceleration to PNN development in DUPs. We suggest that this abnormal response to BDNF represents a developmental pathology in the 16p11.2 DUP syndrome. We note the importance of the MAPKs signaling pathways in the immune and stress responses. The dysregulation of the TAOK-JNK and ERK pathways in DUP neurons may lead to an altered response to environmental factors throughout development, with potential consequences to developmental trajectory. (d) Diagram of our suggested roles of MAPK pathway signaling in GABAergic and PNN development. Our data indicate a role for both the TAOK-JNK and ERK signaling pathways in the regulation of PNN maturation and GABAergic development. However, we found the most dramatic changes to PNN gene expression after modulating JNK, which suggest a specific importance of the JNK family in the regulation of key PNN components. To our knowledge, these results are the first to highlight the link between MAPK signaling and PNN regulation.

Our findings linked JNK, and potentially TAOK2, to the regulation of GABAergic interneuron genes expression. To test TAOK2 involvement, we employed the selective TAOK inhibitor CP-43^68,69^ in WT cortical neurons. We found that TAOK inhibition reduced all pJNK isoforms (supplementary figure S4), most prominently for the smallest JNK isoform (p46) (reduction of around 60% vs vehicle). We assessed whether elevated *Taok2* (TAOK2) and *Mapk3* (ERK1) in 16p11.2 DUP mice relate to the increased PNN density and reduced PVB. In WT cultures treated with either vehicle, ERK inhibitor (PD), or TAOK inhibitor (CP-43), *Acan* expression was reduced with TAOK inhibition. *Sema3a* expression was also reduced when either TAOK or ERK was inhibited (figure 5a). All other PNN components were unchanged with ERK inhibition (figure 5a). However, supporting previous findings where JNK was inhibited, we saw an increase in *Has1* expression with TAOK inhibition (figure 5a).

TAOK inhibition increased *Pvalb* expression (figure 5b), the inverse of observations in DUP mice which overexpress *Taok2*. A modest increase in *Pvalb* expression was found with ERK inhibition. In both ERK and TAOK inhibition conditions, we found reduced *Sst* expression (figure 5b). While *cFos* expression was unchanged when either TAOK or ERK was inhibited, there is some indication of altered network activity, with *Arc* expression markedly lower in both conditions (figure 5b). Furthermore, we found reduced *Dlg4* (PSD-95) expression with both treatments, suggesting the inhibition of these pathways causes aberrant synaptic regulation and altered plasticity.

## Discussion

Our work aimed to understand the mechanisms of BDNF regulation of cortical GABAergic interneuron development and the extent to which they might be compromised in a mouse model of the SZ-related 16p11.2 DUP syndrome.

### BDNF Enhances Cortical GABAergic Maturation in a JNK-Dependent Manner

Our data highlighted an enhancing effect of BDNF on cortical interneuron maturation which supports previous research.^35,70^ BDNF increased the levels of GAD67, WFA labeling intensity, *Kcnc1, Ncan, Bcan,* and *Vcan* expression and reduced embryonic *Gad1* transcript levels, a pattern strongly suggestive of a more mature GABAergic interneuron phenotype. The modulation of key PNN CSPGs by BDNF is important regarding evidence that BDNF controls the closure of the critical period of plasticity in cortical development,^38–40^ since the maturation of PNNs is suggested directly to curtail this plasticity.^31,32,48,64,71,72^

Various studies have highlighted distinct roles for mature and precursor forms of BDNF (mBDNF and proBDNF, respectively).^73^ mBDNF binds TrkB with high affinity but neurotrophin receptor p75^NTR^ with low affinity.^74,75^ Conversely, proBDNF primarily binds p75^NTR^, with lower affinity for TrkB.^76^ We administered only mBDNF in this study, so the enhancing effects of BDNF on GABAergic and PNN maturation are likely driven by mBDNF-TrkB. Given the differing roles of mature and precursor BDNF isoforms, it may also be interesting to investigate the effects of proBDNF on interneuron maturation, and how alterations to the ratios or mBDNF/proBDNF may alter the developmental trajectory of GABAergic neurons.

We found that the enhancing effect of BDNF depends on JNK signaling; the accelerating effects on GAD67 and WFA intensity were negated with JNK inhibition. However, this was not the case when alternative downstream TrkB pathways were inhibited (eg, ERK or PI3K). Moreover, the BDNF upregulation of *Bcan, Ncan*, and *Vcan* was attenuated when JNK was inhibited. These data implicate the JNK pathway in BDNF-TrkB effects on developing GABAergic interneurons.

### JNK Signaling Is Critical for GABAergic and PNN Development

JNK inhibition disrupted PNN development and decreased the density of PNNs at 14 DIV as assessed by WFA labeling. CSPG expression (*Acan*, *Vcan*, *Ncan,* and *Bcan*) were all disrupted after JNK inhibition, and *Sema3a* expression was also reduced. SEMA3A, which binds to CSPGs of PNNs, has a role in axonal outgrowth and neuronal migration and may contribute to PNNs modulation of structural neural plasticity.^58,77^

Conversely, JNK inhibition increased the expression of *Has1*-3. HAS 1-3 are membrane-bound enzymes that synthesize hyaluronan (the backbone upon which CSPGs bind to compose PNNs). HAS1-3 appear to have a dynamic role throughout development, where PNNs undergo extensive maturation and modeling.^78^ Since HAS activity is essential for the formation of the core backbone of new PNNs, we speculate that the upregulation of *Has1-3* in response to JNK inhibition is a compensatory feedback response to the downregulation of CSPGs.

JNK pathway inhibition also reduced GAD65 and GAD dimerization. An SZ-causing *Gad67* mutation impairs GAD dimerization.^79^ Since JNK dysfunction has been implicated genetically and phenotypically with SZ in human cohorts and rodent models,^23,80,81^ the JNK regulation of GAD dimerization may be clinically important.

### 16p11.2 DUP Neurons Exhibit an Abnormal Developmental Trajectory

Basally, the levels of pJNK and pERK were heightened in DUP neurons. GAD levels were lower in DUP cultures vs WTs, while GAD dimerization was elevated. This suggests an overall functional dysregulation of GAD which, given the aforementioned data implicating JNK-signaling in GAD dimerization, may relate to heightened TAOK-JNK activity. BDNF appears to remedy these GAD65/67 deficits, increasing levels to match those in untreated WT cultures. However, GAD dimer levels are not reduced. In DUP neurons, this mechanism does not recruit JNK or ERK, suggesting a reliance on alternative pathways. This may result from elevated basal levels of JNK and ERK, meaning the BDNF response is sequestered to another TrkB pathway.

Additionally, carriers of the 16p11.2 DUP and deletion may have disruption to the PI3K/Akt, as well as MAPK pathways as shown here, due to DUP of the major vault protein (*Mvp*) gene included in the region. MVP regulates the intracellular localization of the protein PTEN, which can antagonize PI3K/Akt.^82,83^ This disruption, coupled with the lack of JNK and ERK response to BDNF shown here, suggests a broadly altered intracellular response to BDNF with the 16p11.2 DUP—potentially a key component of the neurodevelopmental pathology observed in 16p11.2 CNV syndromes. Indeed, BDNF is not only a critical growth factor during development. Since it is also implicated in synaptic plasticity in maturity, altered intracellular responses to BDNF will produce lasting deficits that could contribute to the maintenance of interneuron pathology and symptomology observed in the SZ-related 16p11.2 DUP syndrome.

WFA labeling intensity was notably higher in DUP vs WT-cultured neurons, indicating DUP PNNs reach a more mature density earlier. In P14 PFC of DUP mice, we found heightened expression of *Bcan, Kcnc1, Pvalb,* and mature *Gad1* and lowered expression of a developmentally regulated *Gad1* transcript (Emb *Gad1*) compared with WTs. This further suggests abnormal interneuron development in DUP mice and is a clear indication of precocious GABAergic interneuron development.

This may appear inconsistent with the observed GAD reduction in DUPs, but, considering the pJNK increase in DUP neuronal cultures, it is concurrent with the idea that JNK signaling is crucially important for GABAergic development; in this instance, the development of PNNs. Moreover, in relation to the SZ-related 16p11.2 DUP, this may indicate an abnormally early closure of the critical period of plasticity. The premature closure of this highly plastic window could have profound consequences on the network activity and synaptic connectivity in adulthood.

### 16p11.2 DUP Mice Display GABAergic Interneuron and PNN Abnormalities Into Adulthood

Analysis of adult DUP vs WT PFC revealed that accelerated interneuron development in DUPs, and subsequent upregulation of *Pvalb* and *Gad1*, does not result in a sustained elevation of PVB protein expression in adulthood. Instead, we found that adult DUPs exhibited reduced PVB protein levels in the PFC. This is in line with our previous findings of a decrease in *Pvalb* mRNA expression in the PFC and hippocampus of adult DUP mice.^84^

We speculate that this early closure of the critical period and accelerated development may lead to altered network activity and dynamics, which are sustained into adulthood, and may be mediated by *Pvalb* levels. Our *cFos* data highlight the increased network activity in DUPs adults, which further suggests a pathological disinhibition in these mice. PVB + GABAergic interneurons are known to be the major control mechanism for setting excitation/inhibition balance in the PFC.^85–87^ We theorize that *Pvalb* expression may be downregulated as a compensatory mechanism, in an attempt to restore normal excitation/inhibition balance. A schematic outline of the altered developmental pattern of DUP neurons, along with our suggested mechanisms, is in figures 5c and 5d.

Interestingly, heightened PNN density seems to persist into adulthood in DUP mice, in both PFC and TRN. Our data revealed elevated *Ncan* and *Sema3a* expression in adult DUP mice. Evidence suggests that TAOK2 regulates SEMA3A signaling to JNK,^16^ which may explain the *Sema3a* increase. The *Ncan* increase may indicate this to be a key CSPG, over-produced in DUPs, due to *Taok2* overexpression (and resultant excess JNK activity). Interestingly, *NCAN* maps onto a GWAS-identified SZ locus, and nonsynonymous de novo *NCAN* mutations are enriched in SZ patients.^88^ Together, these findings implicate disrupted *Ncan* expression and function in SZ pathology, which may extend beyond carriers of the 16p11.2 DUP and affect a wider subset of SZ patients.

Curiously, in the TRN, both WFA and PVB intensity appear to be slightly elevated in comparison to WT. These data indicate a complex relationship between PNN density and PVB expression and may suggest a divergence of the mechanisms controlling GABAergic interneuron development and regulation across various brain regions.^89–91^

### TAOK2-JNK Signaling as a Key Regulatory Pathway in Maturation of GABAergic Interneurons and PNNs

Until now it has been unclear how SZ genetic risk factors are connected to the characteristic impairments in PFC PVB cells. In WT neurons, TAOK inhibition increased the *Pvalb* expression. This is pertinent considering the PVB reduction in adult DUP PFC (*Taok2* overexpression). It suggests a mediatory role for Taok2 in *Pvalb* expression. We did not find the same increase in *Pvalb* expression when ERK was inhibited.

Importantly, while ERK signaling affects some aspects of the GABAergic phenotype (eg, *Sst* expression), ERK inhibition did not dramatically affect PNN gene expression. TAOK inhibition modulated the expression of key PNN components, *Sema3*a, *Acan*, and *Has1*, and altered *Pvalb* and *Sst* expression. However, JNK inhibition produced the most dramatic effect on PNN-related genes, downregulating all CSPGs and *Sema3a*, while upregulating *Has1-3* expression. This suggests that, while other MAPKs may be involved to some extent, the TAOK2-JNK signaling cascade is a key regulatory pathway in GABAergic interneuron and PNN development. In fact, given the magnitude of the effects of JNK inhibition on CSPGs, we suggest a major role for JNK signaling specifically in PNN regulation. An outline of our suggested role for MAPK pathways (TAOK-JNK and MEK-ERK) in the developmental control of cortical GABAergic interneurons and PNNs is summarized in figures 5c and 5d.

Accumulating evidence suggests that dysfunctional JNK activity may be a substantial contributor to SZ risk. It is clear that cortical interneuron dysfunction accounts for some of the pathology and symptomology observed in SZ. Understanding the regulators of the development of these interneurons is clearly important. These data, implicating TAOK2-JNK signaling directly in adverse development of PFC GABAergic interneurons and PNNs in SZ, highlight a promising novel therapeutic strategy for exploration.

## Funding

This research was supported by the Medical Research Council (UK) (grant MR/N012704/1).

## Supplementary Material

sbaa139_suppl_Supplementary_Material
