# Supplementary material for: BDNF and JNK Signaling Modulate Cortical Interneuron and Perineuronal Net Development: Implications for Schizophrenia-Linked 16p11.2 Duplication Syndrome
Source: Schizophr Bull. 2020 Oct 17;47(3):812–26. doi: 10.1093/schbul/sbaa139 (PMC8084442; doi:10.1093/schbul/sbaa139)
Supplement: sbaa139_suppl_Supplementary_Material [file sbaa139_suppl_supplementary_material.pdf]

Supplementary Material

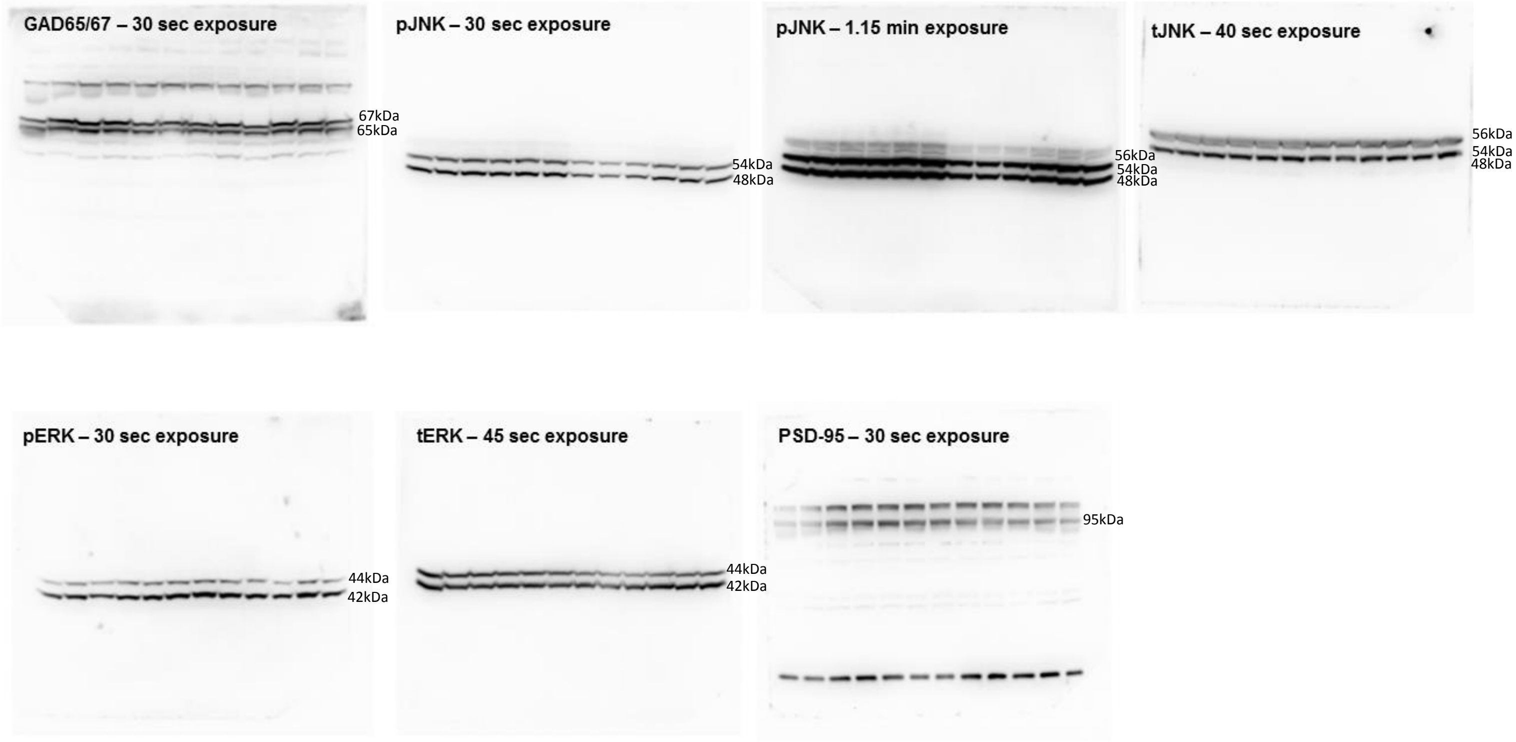

Figure S1. Example images of full western blot membranes for all antibodies used in this study.

25µl/lane (~2.5µg/µl) of protein samples were prepared with 4x sample buffer (NuPAGE, Novex, NP0007) and sample reducing agent (NuPAGE, Novex, NP0004). Protein was denatured at 80°C for 10min. Samples were then subjected to SDS-PAGE in 10 % Bis-Tris gel (NuPAGE, Novex, NP0302BOX) run at 120 volts for 2 hours in chilled buffer and transferred to Invitrolon PVDF membranes (Novex, LC2005) (30 volts, 1 hour). Following transfer, membranes were washed twice in dH_2O and blocked 0.5% Tween-tris-buffered-saline (TTBS) + 3% dried milk for 30 min at room temperature. Membranes were then incubated overnight at 4°C with target antibody diluted in either TTBS/1% milk or SignalBoost Immunoreaction Enhancer solution (Millipore, 407207). The following morning, membranes were washed 3 times (10 min) in TTBS and incubated in HRP-conjugated anti-rabbit secondary antibody (1:10,000, supplemented with 1% dried milk) for 2 hours at room temperature. Membranes were washed again, once with TTBS and then twice in 10X Tris-Buffered Saline for 10 minutes prior to imaging. Blots were probed for a second time using GAPDH-HRP antibody. Membrane-bound antibodies were detected on the blot with a Chemiluminescent HRP Substrate (Immobilon, Millipore, WBKLS0100), and digital images were captured via PXi4 (Syngene). Exposure times are indicated on blot images.

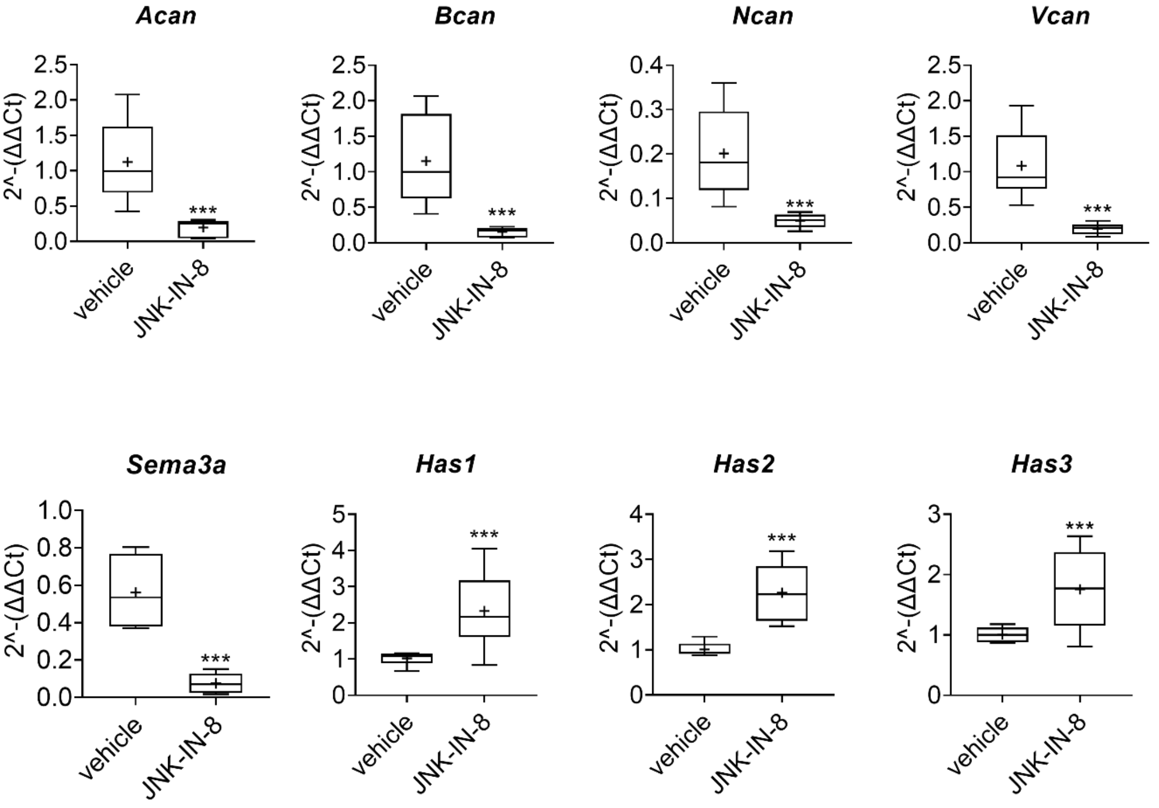

Figure S2. JNK inhibition decreased mRNA expression of key PNN CSPGs but elevated expression of hyaluronan synthase.

mRNA expression of key genes related to the structure and function of PNNs were assessed via qPCR in 21DIV primary neuronal cultures treated with vehicle or JNK-IN-8 (5 DIV prior to extraction; ie. at 16 DIV). The later time-point chosen for inhibition (16 – 21 DIV) reflected the low levels or later developmental onset of some target genes (e.g. Acan) and the subsequent requirement to have culture extracts containing high enough expression for qPCR detection. None-the less, our pharmacological manipulation was implemented well in advance of PNN maturity and during a key developmental time-point for many interneuron subtypes for an extended period (e.g. PVB interneurons, of which expression isn't measurable until 21DIV). Genes included (a) CSPGs (*Ncan, Bcan, Acan, Vcan*)*, Sema3a* and hyaluronan synthases (*Has1-3*). Notably, JNK inhibition dramatically reduced the expression of all CSPGs and Sema3a. Conversely, *Has1-3* expression was upregulated in response. Relative expression was calculated using ΔΔCt method with *Tbp* housekeeping gene. For boxplots: Centre lines show the medians; box limits indicate the 25th and 75th percentiles; whiskers extend 1.5 times the interquartile range from the 25th and 75th percentiles; crosses indicate sample means. Data were analysed via one-way ANOVA with Tukey post-hoc multiple comparisons. *** represents Tukey post-hoc significance compared to vehicle ($p<0.01$). n= 6 independent samples per condition.

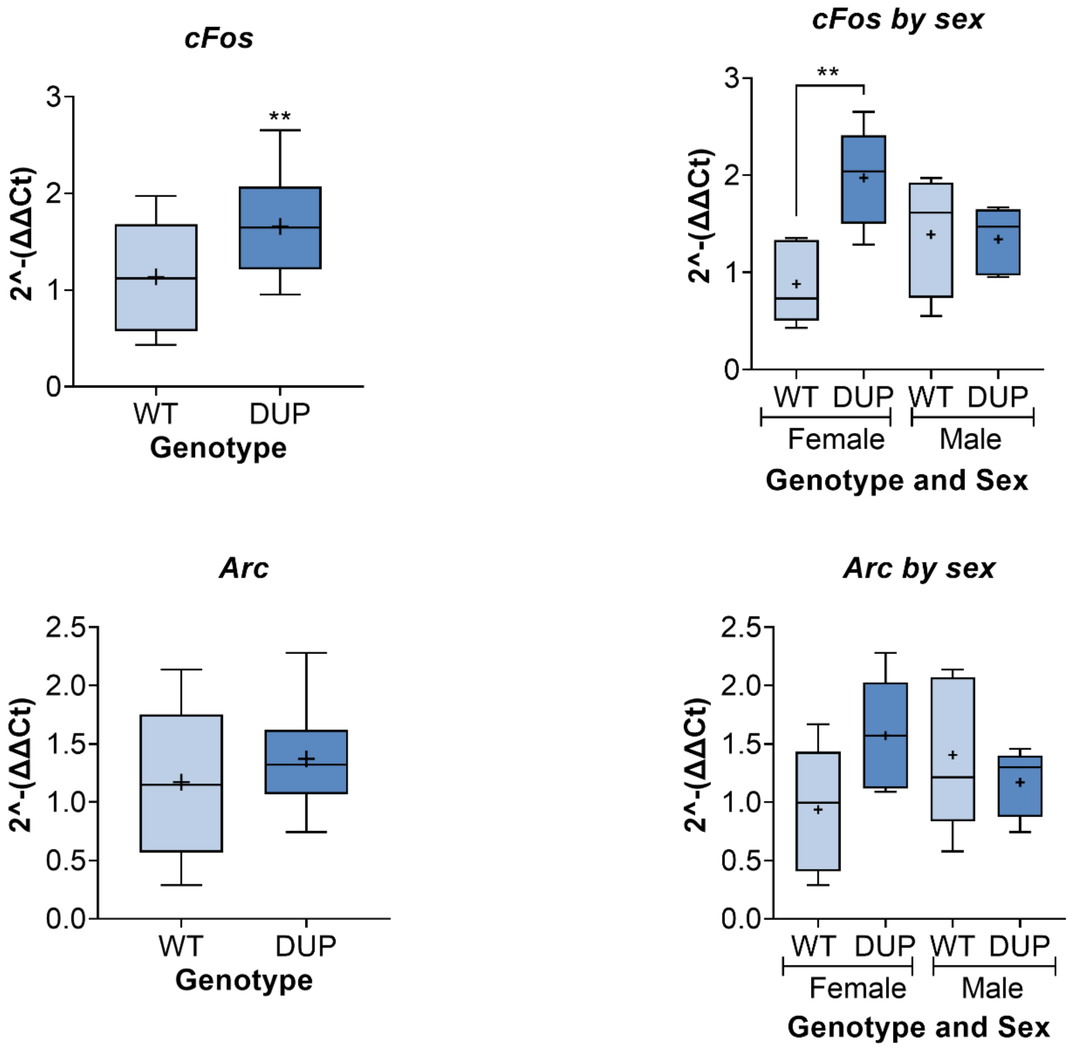

Figure S3. Elevated cFos expression in adult 16p11.2 duplication vs. WT PFC. We found a significant increase in DUP *cFos* expression compared to WT. Upon further examination, our ANOVA revealed a significant genotype-sex interaction. In contrast, we found no clear difference between *Arc* expression in WT vs. DUPs. Relative expression was calculated using ΔΔCt method with *Gapdh* housekeeping gene. Data were analysed via two-way ANOVA with genotype and sex as factors; factors were crossed to assess any existent genotype-sex interaction. ** represents Tukey post-hoc significance ($p<0.05$). N=10 per genotype (5 female, 5 male per genotype).

.

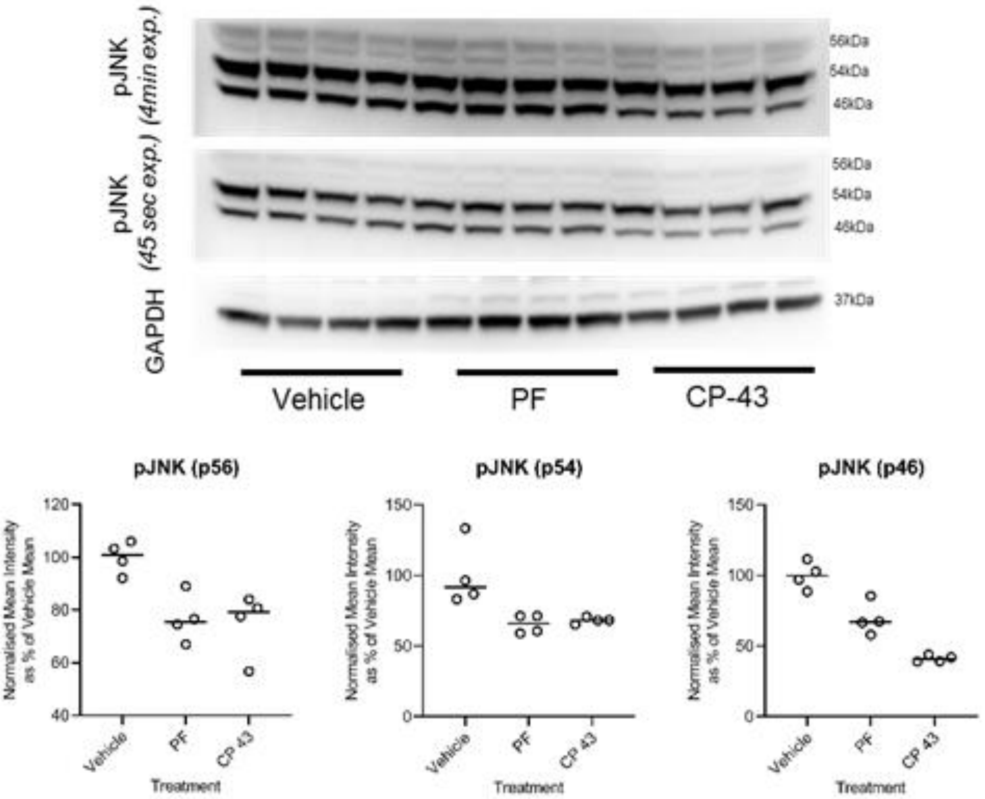

Figure S4. TAOK inhibition reduced pJNK levels in WT primary cultured neurons measured via western blot. Cultures were treated at 14 DIV with either vehicle, PF06260933 (20µM; MAP4K4) inhibitor, [1, 2]) or CP-43 (30µM; TAOK inhibitor) for 3 hours before protein was extracted.

Table 1. Table of antibodies used in the study. (a) Primary antibodies (b) fluorescent secondary antibodies.

a

**Antibody**	**Species**	**Dilution**	**Company**
GAD65/67	Rabbit	ICC: 1:5000, WB: 1:8000	Sigma
Biotinylated WFA lectin		ICC: 1:2000, IHC: 1:1000	Vector Laboratories
PVB	Mouse	IHC: 1:1500	Sigma
pJNK	Rabbit	WB: 1:12000	Abcam
tJNK	Rabbit	WB: 1:10000	Cell Signalling Technologies
pERK	Rabbit	WB: 1:5000	Cell Signalling Technologies
tERK	Rabbit	WB: 1:5000	Cell Signalling Technologies
PSD-95	Rabbit	WB: 1:2000	GeneTex
GAPDH-HRP conjugated	Rabbit	WB: 1:20000	GeneTex

b

Fluorescent Secondary Antibodies	Target	Host	Dilution	Company
Alexa Fluor 488	Mouse	Donkey	IHC: 1:500	Jackson ImmunoResearch
Alexa Fluor 488	Rabbit	Donkey	IHC: 1:500, ICC: 1:300	Jackson ImmunoResearch
Steptavidin-conjugated Rhodamine Red-X			IHC: 1:1000, ICC: 1:500	Jackson ImmunoResearch

Table S2. Primer sequences used for RT-qPCR.

Target Gene	Forward Primer Sequence (5'->3')	Reverse Primer Sequence (5'->3')
Acan	GTTGCAGACATTGACGAGTGC	AGTCCACCCCTCCTCACATT
Bcan	GATTCCGGGGTCTATCGCTG	ACGACCCCTTTGACCTTGAC
Ncan	AGTATGGGGGCCGGATCTGT	TGGTGTCCTGTGTGTCCTGAT
Vcan	ACCTTCCAACTATCCGGTGC	GGTATGCAGATGGGTTCATGC
Has1	TGTGTCCTGCATCAGTGGTC	TTGGTGAGGTGCCTGTCATC
Has2	GCCATGTGGTTTCACAAGCA	TGAGACCCACTAGCTGGACA
Has3	GCTTCTTTGTGTGGCGTAGC	AGTCCACTGAGTTGCCAAGG
Sema3a	GCCTGTCTTTTCTGGGGTGT	AGCTGTTGGCCAAGCCATTA
Dlg4	CTCAAGAGGCGGGTTCAT	AAGCCAAGTCCTTTAGGCCC
Sst	CCAACTCGAACCCAGCAATG	TCAGAGGTCTGGCTAGGACA
Pvalb	CAAGCAGTCAGCGCCACTTA	GGATGAGCTGGGGTCCATTCT
cFos	TTTCAACGCCGACTACGAGG	GCGCAAAAGTCCTGTGTGTT
Arc	GAGCGAGAGCTGAAAGGGTT	ACGGTAGAAGACCTCCCTCC
Gad1 (mature)	TTTGGAGCTGTCTGACCACC	AAATCGAGGGTGACCTGTGC
Embryonic Gad1	GTGGCCTCCAGAGGTTCACAT	CTCCCCCAGGAGAAAATATCCC
Kcnc1	ACTCAGAGTGACACATGCCC	CCCATTCAGTTTGGAATCTGCT
Pax6	CGGATGAAGCTCAGATGCGA	CTCAAACTCTTTCTCCAGAGCCT
Gapdh	AATGTGTCCGTCGTGGATCT	AGACAACCTGGTCCTCAGTG
Tbp	TGCTGTTGGTGATTGTTGGT	AACTGGCTTGTGTGGGAAAG

Table S3. F, T and p-values from statistical tests between experimental groups.

A. Western blot and ICC analysis of WT cultured neurons treated with vehicle, inhibitor (SP, Wortmannin or PD), BDNF + inhibitor. Analysed via three-way ANOVA (factors: BDNF, inhibitor, culture batch. BDNF and inhibitor crossed to assess any interaction). Data and Tukey post-hoc comparisons are presented in Fig.1.

Experiment	Target Protein	Main effect of BDNF	Main effect of inhibitor	BDNF x inhibitor interaction
**BDNF - JNK inhibition**	GAD65	p=0.001, $F_{(1,35)}$=13.01	p=0.005, $F_{(1,35)}$=9.13	p=0.006, $F_{(1,35)}$=8.91
	GAD67	p<0.000, $F_{(1,35)}$= 18.25	p<0.000, $F_{(1,35)}$=18.23	p<0.000, $F_{(1,35)}$=16.60
	GAD dimer	p=0.001, $F_{(1,35)}$=12.36	p<0.000, $F_{(1,35)}$=20.24	p=0.003, $F_{(1,35)}$=10.45
	WFA	p<0.001, $(_{F1, 982})$ = 45.46	p<0.001, $(_{F1, 982})$ = 137.57	p<0.001, $F_{(1, 982)}$ = 19.09
**BDNF - PI3K inhibition**	GAD65	p= 0.002, $F_{(1,35)}$ = 10.94	p= 0.978, n.s	p=0.043, $F_{(1,35)}$=4.47
	GAD67	p<0.000, $F_{(1,35)}$= 25.21	p=0.434, n.s	p=0.270, n.s
	GAD dimer	p<0.000, $F_{(1,35)}$=18.69	p=0.001, $F_{(1,35)}$=14.55	p=0.003, $F_{(1,35)}$=10.62
**BDNF - ERK inhibition**	GAD65	p=0.017, $F_{(1, 35)}$=6.37	p=0.440, n.s	p=0.072, n.s
	GAD67	p=0.001, $F_{(1,35)}$=13.45	p=0.734, n.s	p=0.634, n.s
	GAD dimer	p=0.0.029, $F_{(1,35)}$=5.24	p=0.569, n.s	p=0.126, n.s

B. qPCR analysis of WT cultured cortical neurons treated with vehicle, JNK-IN-8, BDNF or JNK-IN-8 + BDNF at 14 DIV for 7 DIV. Data analysed via two-way ANOVA (factors: BDNF and JNK-IN-8, with BDNF and JNK-IN-8 crossed to investigate the presence of any significant interaction). Additionally, Mann Whitney U-Tests were performed for *Sema3a* and *Has2.* Data presented in Fig. 2a

Target Gene	Main effect of BDNF	Main effect of JNK-IN-8	BDNF x JNK-IN-8 interaction
Acan	p<0.000, $F_{(1,23)}$=23.29	p=0.014, $F_{(1,23)}$=7.32	p=0.194, n.s
Bcan	p=0.06, $F_{(1,23)}$=3.99	p<0.000, $F_{(1,23)}$=140.69	p=0.005, $F_{(1,23)}$=9.81
Ncan	p=0.385, n.s	p<0.000, $F_{(1,23)}$=41.15	p=0.016, $F_{(1,23)}$=6.94
Vcan	p=0.004, $F_{(1,23)}$=10.85	p<0.000, $F_{(1,23)}$=85.16	p=0.011, $F_{(1,23)}$=7.94
Sema3a	p=0.002, $F_{(1,23)}$=12.77	p=0.500, n.s	p=0.201, n.s
Has1	p=0.064, $F_{(1.23)}$=3.83	p<0.000, $F_{(1,23)}$=18.92	p=0.183, n.s
Has2	p=0.735, n.s	p=0.092, n.s	p=0.125, n.s
Has3	p=0.215, n.s	p=0.002, $F_{(1,23)}$=12.14	p=0.139, n.s

Target	Mann Whitney U-Test
Sema3a	Veh vs. JNK-IN-8: p=0.0325
Has2	Veh vs. JNK-IN-8: p=0.0660
Has3	Veh vs. BDNF: p=0.004

C. qPCR analysis of maturity related genes in cultured cortical neurons treated with either vehicle or BDNF at 14 DIV for 7 DIV. Data were analysed via independent sample t-tests. Data presented in Fig. 2b.

Target Gene	
Kcnc1	t(10)=2.67, p=0.047
Pax 6	t(10)= 2.40, p=0.07

Emb Gad1	t(10)= 1.96, p=0.038

D. Protein expression and PNN density WT vs. DUP cultured neurons. Data were analysed via three-way ANOVA (factors: genotype, treatment, culture batch. Factors genotype and treatment were crossed to investigate the existence of any significant interactions). Data and Tukey post-hoc multiple comparisons are presented in Fig. 3.

**Target Protein**	**WT vs. DUP (effect of genotype)**	**WT vs. DUP (effect of treatment)**	**genotype x treatment interaction**
pJNK (p46)	p=0.091, n.s	p=0.031, $F_{(1,71)}$=4.83	p<0.000, $F_{(1,71)}$=21.80
pJNK (p54)	p=0.019, $F_{(1,71)}$=5.76	p=0.012, $F_{(1,71)}$= 6.65	p=0.002, $F_{(1,71)}$=10.39
pJNK (p56)	p=0.075, n.s	p=0.074, n.s	p<0.000, $F_{(1,71)}$=22.85
pERK (p42)	p<0.000, $F_{(1,59)}$=20.16	p=0.006, $F_{(1,59)}$=8.05	p<0.000, $F_{(1,59)}$=22.71
pERK	p=0.090, n.s	p=0.032, $F_{(1,59)}$=4.85	p=0.011, $F_{(1,59)}$=6.97
GAD65	p=0.001, $F_{(1,71)}$=12.12	p<0.000, $F_{(1,71)}$=14.08	p=0.084, n.s
GAD67	p=0.014, $F_{(1,71)}$=6.32	p<0.000, $F_{(1,71)}$=19.46	p=0.848, n.s
GAD Dimer	p=0.004, $F_{(1, 35)}$=9.72	p=0.416, n.s	p=0.993, n.s
PSD-95 - Upper/Lower Ratio	p=0.015, $F_{(1,23)}$=7.18	p=0.018, $F_{(1.23)}$=6.77	p=0.324, n.s
WFA	p=0.010, $F_{(1, 35)}$=7.50	p=0.012, $F_{(1,35)}$=7.20	p=0.556, n.s

E. qPCR analysis of WT vs. DUP P14 PFC. Data were analysed via independent sample t-tests. Data presented in Fig. 3l-n.

**Target Gene**	
Acan	t(14)= 1.088, p=n.s
Bcan	t(14)= 3.221, p=0.0062
Ncan	t(14)= 0.4567, p=n.s
Vcan	t(14)= 1.076, p=n.s
Gad1 (mature)	t(14)= 2.220, p=0.0434
Pvalb	t(14)= 2.554, p=0.0229
Kcnc1	t(14)= 2.656, p=0.0188
EmbGad1	t(14)= 5.556, p=0.0037

F. WT vs. DUP WFA and PVB staining intensity in adult PFC and TRN. Data were analysed via three way ANOVA (factors: genotype, sex, pair. Genotype and sex were crossed to investigate any potential interactions). Data and Tukey post-hoc multiple comparisons are presented in Fig.4 a-d.

**Target Protein**	**Region**	**WT vs. DUP (effect of genotype)**	**WT vs. DUP (effect of sex)**	**genotype x interaction**
WFA	TRN	p<0.000, $F_{(1,1429)}$= 123.28	p<0.000, $F_{(1, 1429)}$= 12.25	p<0.000, $F_{(1,1429)}$=15.26
PVB	TRN	p<0.000, $F_{(1,1754)}$= 16.42	p<0.000, $F_{(1,1754)}$= 15.12	p<0.000, $F_{(1,1754)}$= 127.20
WFA	PFC	p<0.000, $F_{(1,1546)}$=128.35	p=0.906, n.s	p=0.130, n.s
PVB	PFC	p<0.000($_{F1, 821}$)=497.79	p=0.004, $F_{(1,821)}$= 8.33	p=0.678, n.s

G. mRNA expression WT vs DUP adult PFC. Data were analysed via two-way ANOVA (factors genotype and sex, with factors crossed to assess the presence of any significant interactions). Data presented in Fig. 4e and Fig. S3.

Target Gene	WT vs. DUP (effect of genotype)	WT vs. DUP (effect of sex)	genotype x sex interaction
Acan	p=0.766, n.s	p=0.076, n.s	p=0.770
Bcan	p=0.821, n.s	p=0.260, n.s	p=0.860, n.s
Ncan	p=0.003, $F_{(1,19)}$=11.79	p=0.038, $F_{(1,19)}$=5.13	p=0.909, n.s
Vcan	p=0.815, n.s	p=0.53, $F_{(1,19)}$=4.35	p=0.231, n.s
Sema3a	p<0.000, F $_{(1,19)}$=15.21	p=0.573, n.s	p=0.821, n.s
Has1	p=0.154, n.s	p=0.132, n.s	p=0.105, n.s
Has2	p=0.633, n.s	p=526, n.s	p=0.388, n.s
Has3	p=0.720, n.s	p=0.042, $F_{(1,19)}$=4.88	p=0.212, n.s
cfos	p=0.029, F(1,19)=5.75	p=0.782, n.s	p=0.019. $F_{(1,19)}$=6.86
Arc	p=0.396, n.s	p=0.886, n.s	p=0.075, n.s

H. qPCR analysis of WT cultured neurons treated with either vehicle, PD or CP-43. Data were analysed via one-way ANOVA. Additionally, Mann Whitney U-Tests were performed for *Acan* and *Has1*. Data presented in Fig. 5.

Target Gene	Effect of treatment
Acan	p=0.059, $F_{(1,23)}$=3.28
Bcan	p=0.136, n.s
Ncan	p=0.727, n.s
Vcan	p=0.133, n.s
Sema3a	p<0.000, $F_{(1,23)}$=25.63
Has1	p=0.078, n.s
Has2	p=0.365, n.s
Has3	p=0.303, n.s
cfos	p=0.850, n.s
Arc	p<0.000, $F_{(1,23)}$=13.52
Pvalb	p=0.043, $F_{(1,23)}$=3.76
Sst	p<0.000, $F_{(1,23)}$=18.05
Dlg4	p=0.002, $F_{(1,23)}$=8.17

I. qPCR analysis of WT cultures treated with vehicle or JNK-IN-8 after 5 DIV treatment (16-21 DIV). Data were analysed via one-way ANOVA. Data presented in Fig. S2.

Target Gene	Effect of treatment
Acan	p=0.003, F(1,11)=14.72
Bcan	p=0.003, F(1,11)=14.61
Ncan	p=0.004, F(1,11)=13.33
Vcan	p=0.001, F(1,11)=18.85
Sema3a	p=0.01, F(1,11)=31.42
Has1	p=0.015, F(1,11)=8.67

Has2	p=0.002, F(1,11)=18.28
Has3	p=0.022, F(1,11)=7.39
